# Flight behaviour of malaria mosquitoes around odour-baited traps: capture and escape dynamics

**DOI:** 10.1098/rsos.180246

**Published:** 2018-08-08

**Authors:** Antoine Cribellier, Jens A. van Erp, Alexandra Hiscox, Martin J. Lankheet, Johan L. van Leeuwen, Jeroen Spitzen, Florian T. Muijres

**Affiliations:** 1 Experimental Zoology Group, Wageningen University, Wageningen, The Netherlands; 2 Laboratory of Entomology, Wageningen University, Wageningen, The Netherlands

**Keywords:** *Anopheles coluzzii*, insect flight behaviour, host-seeking, avoidance manoeuvres, trap efficiency, vector control

## Abstract

Host-seeking mosquitoes rely on a range of sensory cues to find and approach blood hosts, as well as to avoid host detection. By using odour blends and visual cues that attract anthropophilic mosquitoes, odour-baited traps have been developed to monitor and control human pathogen-transmitting vectors. Although long-range attraction of such traps has already been studied thoroughly, close-range response of mosquitoes to these traps has been largely ignored. Here, we studied the flight behaviour of female malaria mosquitoes (*Anopheles coluzzii*) in the immediate vicinity of a commercially available odour-baited trap, positioned in a hanging and standing orientation. By analysing more than 2500 three-dimensional flight tracks, we elucidated how mosquitoes reacted to the trap, and how this led to capture. The measured flight dynamics revealed two distinct stereotypical behaviours: (i) mosquitoes that approached a trap tended to simultaneously fly downward towards the ground; (ii) mosquitoes that came close to a trap changed their flight direction by rapidly accelerating upward. The combination of these behaviours led to strikingly different flight patterns and capture dynamics, resulting in contrasting short-range attractiveness and capture mechanism of the oppositely oriented traps. These new insights may help in improving odour-baited traps, and consequently their contribution in global vector control strategies.

## 1. Introduction

Haematophagous insects need blood meals for reproduction. As a result, they have to interact with vertebrate hosts that have various defence strategies and that are sometimes even their predators. Thus, mosquitoes need to minimize the risk induced by this interaction with their host by feeding quickly, stealthily and effectively [1,2]. Additionally, when approaching a host, mosquitoes must be aware of any cues announcing host defensive behaviours such as looming objects or air gusts. Surprisingly, the impact of such disruptive cues on the short-range attraction of anthropophagic mosquitoes towards human hosts has been studied very little [3,4]. Actually, mosquito flight dynamics in reaction to humans or objects imitating hosts also received little attention [5].

By contrast, the odour-mediated host-seeking behaviour of mosquitoes has been studied extensively, because it is relevant for mosquito population management (vector control). Anthropophilic female mosquitoes are attracted to their hosts by a species-specific cocktail of human odours and CO_2_ [6–8]. When a host-seeking mosquito encounters these cues, it performs a stereotypical ‘cast and surge’ flight behaviour in order to locate the host [6,9,10]. Similar flight behaviour is observed in many species of insects, such as moths, fruit flies and mosquitoes to find potential mates, food or hosts, respectively [11–13].

Detailed studies on host-seeking behaviour showed that flying *Aedes aegypti* mosquitoes (vectors of e.g. dengue and Zika fever) use a combination of cues to find and approach hosts [6,14,15]. At long distances, they only use CO_2_ and odours to find a potential host via ‘cast and surge’ flights. At intermediate distances (of the order of metres), they start to inspect visual cues (highly contrasting objects) near the ground. And at short ranges (less than 1 m), they use heat and moisture cues to find a landing spot on the host [7,9,16,17]. One could suppose that the relatively high importance of visual cues in *Aedes aegypti* arises from their diurnal behaviour, but recent studies on host-seeking *Anopheles* mosquitoes shows that these nocturnal mosquitoes have a similar reaction to visual cues under moonlit or starlit conditions [18,19].

**Figure 1.**
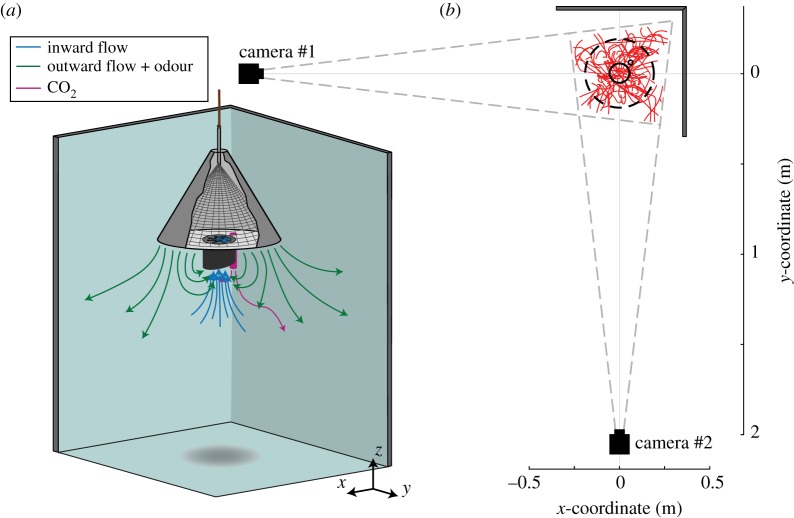
The experimental set-up. (*a*) Experimental set-up with floor, side-walls and a hanging Suna trap. A slice removed from the circular pyramid makes the inside of the trap visible. The fan of the trap (blue) creates a circulating airflow that attracts mosquitoes by pushing air mixed with the human-odour mimicking MB5 blend away from the trap (green arrows), and captures mosquitoes by sucking air (blue arrows) into the main entry tube (black). Mosquitoes are then confined inside by a net and by a trap door that closes when the fan is not working. A pipe (purple) releases CO_2_ to simulate human breath. Arrows illustrate how the trap used airflow to attract and capture mosquitoes. (*b*) Top-down view of the experimental set-up including the two high-speed video cameras, placed perpendicular to one another at 2 m from the trap centre. The filmed region, near the two background walls, is limited by the angle of view of each camera (grey dotted lines). The flight tracks recorded during a 15 min session (with a standing trap) are shown in red as an example. The outflow platform of the trap, its entrance and its CO_2_ pipe are represented by the dashed circle and the large and small solid circles, respectively.

Based on the developed knowledge of host-seeking behaviour in mosquitoes, a range of different mosquito traps has been developed [20–22]. One promising odour-baited trap type is the counter-flow trap that uses a single fan to generate both an inward airflow for capturing mosquitoes as well as an outward directed airflow carrying attractive odour away from the trap (figure 1*a*). The odour bait consists of a combination of chemicals mimicking human skin odour. Trap models usually combine the odour bait with CO_2_ and/or visual cues in contrasting black and white, such as the BG-Sentinel trap and the BG-Suna trap (Biogents AG, Regensburg, Germany) [23,24]. Although odour-baited traps were originally developed as research tools [25], a recent large-scale field study in Kenya showed that in combination with pre-existing bed nets, odour-baited traps reduced the number of human malaria cases by 30% [26]. This study indicated that the use of such insecticide-free traps could now be considered as an effective supplement to conventional vector control systems.

By combining multiple host cues such as odours, CO_2_ and visual contrast, odour-baited traps aim to trigger the natural host-seeking behaviour of mosquitoes. But surprisingly little attention has been given to the optimization of the capture mechanism of the trap. This might, at least partly, be due to a lack of detailed knowledge on the flight dynamics of mosquitoes in the vicinity of traps. In addition, until now, mosquito traps have been primarily optimized via an iterative design process, where the number of mosquitoes caught is compared between different trap designs [25,27,28]. Thus, obtaining detailed knowledge of mosquito flight dynamics around traps should open new paths for trap improvement and could be crucial in identifying the short-range approach and capture mechanisms involved [29].

To the best of our knowledge, Cooperband & Cardé [5] are the only ones to have studied mosquito flight behaviours near traps. They analysed three-dimensional tracks of *Culex quinquefasciatus* and *Culex tarsalis* approaching four models of CO_2_-baited traps in a large field wind tunnel, and showed that the traps had very different capture efficiency (4–26% of upstream released mosquitoes were captured). In addition, they highlighted that, when approaching the trap, mosquitoes decelerated and adopted tortuous flights that varied in dynamics between the traps. These differences in dynamics, especially in the close vicinity of the traps, might help explain the differences in capture rate among the traps, but the spatial and temporal resolution of their flight tracking system did not allow for a detailed analysis of these dynamics [5].

Here, we will zoom in on these tortuous flight manoeuvres close to the trap to elucidate how the close-range response of the mosquitoes affects their capture dynamics. These close-range dynamics complement the long-distance approach dynamics as previously studied by Cooperband & Cardé [5]. We specifically address the tortuous flight paths close to the trap, and how these flight manoeuvres eventually lead to capture or escape from the trap.

**Figure 2.**
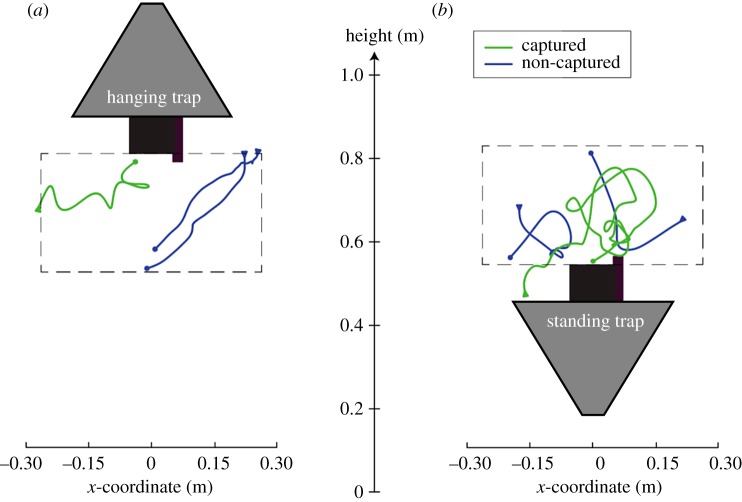
Examples of flight tracks around the two traps. Examples of three flight tracks around the hanging trap (*a*) and standing trap (*b*), as viewed by camera #2. Tracks that lead to capture are in green and tracks from non-captured mosquitoes are in blue. The start and end of each track are represented by an arrowhead and a dot, respectively. Videos of these flight tracks can be found in the electronic supplementary material, videos S1 and S2. The grey dashed line indicates the cylindrical volume for which we plotted the distribution of various flight parameters in the form of heat maps (figures 4, 6 and 8).

For this study, we used female malaria mosquitoes *Anopheles coluzzii*, and the odour-baited BG-Suna trap (Biogents AG), which was developed and is used for malaria vector control in Africa [26]. We tested the trap in two orientations, the original hanging orientation and an upside-down standing orientation. The trap in its original hanging orientation has an upward-directed airflow for capturing mosquitoes (figure 2*a*), whereas the upside-down standing trap has a downward-directed airflow for mosquito capture, and thus simulated the widely used and similar BG-Sentinel trap (figure 2*b*) [23,24]. The use of these two trap orientations allowed us to investigate whether mosquitoes exhibit distinct behaviours in response to odour cues and opposite airflow orientations, and how this affects the capture dynamics.

We filmed a total of 530 mosquitoes flying around the trap using a stereoscopic high-speed videography system, from which we reconstructed more than 2500 three-dimensional flight paths. Based on these results, we found that the tortuous flight behaviour of mosquitoes near odour-baited traps was the result of two distinct and stereotypic behavioural responses of the mosquitoes to the trap: (i) when a mosquito flew towards the trap, it would tend to simultaneously fly downwards towards the floor, possibly in order to host-seek near the ground; (ii) when a mosquito came close to the trap, it responded to the strong air currents induced by the trap or to a lack of short-range host cues, by performing an upward-directed manoeuvre, leading to high vertical accelerations in the flight path. Similar behaviours have been described previously in the literature [9,18,30–32]. Here we showed that the combination of these two stereotypical behaviours can lead to strikingly different flight dynamics depending only on trap orientation, which consequently led to similar differences in capture dynamics and capture efficiencies between the traps. These new insights into the close-range interaction between mosquito and trap may help in improving future trap designs.

## 2. Material and methods

### 2.1. Experimental animals

For this study, we used a colony of *Anopheles coluzzii* (from the *Anopheles gambiae* complex [33]) that originated from Suakoko, Liberia in 1987. Since then, the colony has been reared at the Laboratory of Entomology (Wageningen University & Research, The Netherlands) at a temperature of 27°C, a relative humidity of 70%, and with a clock shifted 12 L : 12 D cycle. Adults were kept in BugDorm cages (30 × 30 × 30 cm) in which they were fed on sugar-water with 6% glucose solution and offered daily blood meals from a blood bank (Sanquin, Nijmegen, The Netherlands) via a membrane feeding system (Hemotek, Discovery Workshop, UK). Thereafter, they were allowed to lay eggs on wet filter paper, which were then moved to a plastic tray filled with water. Liquifry No.1 fish food and TetraMin Baby were provided for larvae feeding. Finally, pupae were placed in new BugDorm cages to emerge. During our experiments, we used non-blood fed female malaria mosquitoes, which had most likely mated (5–10 days post-emergence with males and females housed together). They were collected between 12 and 16 h before experiments and were not blood fed in order to increase host-seeking responses.

### 2.2. Experimental set-up

Experiments were conducted in a 3.01 × 4.92 × 3.25 m (width × length × height) climate room at the Laboratory of Entomology (Wageningen University & Research, The Netherlands). The room was maintained at 27°C and 70% relative humidity and had a continuously running air filter system preventing accumulation of odours or CO_2_ (see Spitzen *et al*. [10] for more details). The experimental set-up consisted of a stereoscopic high-speed camera system that filmed the vicinity of a BG-Suna trap (Biogents AG) positioned in front of two perpendicular walls, with the trap centre at 35 cm from each wall (figure 1). A net covered a volume of approximately 8 m^3^ around the set-up in order to prevent mosquitoes from escaping.

The BG-Suna trap is an odour-baited trap with a diameter of 52 cm and height of 39 cm that was developed for malaria vector control in Africa [26]. For each separate experimental trial, the trap was randomly positioned in its original hanging orientation (figure 2*a*), or in a standing orientation in which it resembled a BG-Sentinel trap (figure 2*b*) [23,24]. In its original hanging orientation, the trap produced an upward-directed airflow for capture, whereas the upside-down standing trap produced a downward-directed airflow for mosquito capture. The capture entrance of the hanging and standing trap had a respective height of 81 cm and 54.5 cm above ground level (figure 2*a,b*, respectively). These heights were chosen to keep the camera heights equal to 62 cm in order to avoid repetitive realignment and recalibration of the camera system. Note that because of this reason, the hanging trap was positioned higher than the 30 cm height that has been found optimal for capturing mosquitoes [25].

We used the MB5 blend of five attractants [34] inside each trap to simulate human odour. The CO_2_ release pipe of the trap was connected to a pressurized gas canister containing a mixture of 5% CO_2_ + 95% air and with a flow rate of 200 ml min^–1^. To minimize blind spots in the camera views, this CO_2_ pipe was shortened by 3.25 cm relative to the original length for the Suna trap. All handling of the materials and mosquitoes was done wearing nitrile gloves to minimize the risk of skin odour contamination of the traps.

Because *Anopheles* mosquitoes are night active, we performed the experiments in dimmed light conditions, using a single spotlight (15 W incandescent bulb) directed towards the ceiling, and experiments were carried out during the period of the day which corresponded to the dark phase of the mosquito rearing; the period when the *An. coluzzii* were expected to exhibit the greatest degree of host-seeking behaviour.

Mosquito tracks around the trap were recorded using two synchronized high-speed cameras (PROMON 501 camera head with NIR sensors and 45 mm lenses), filming at 90 frames per second with a resolution of 1240 × 1080 pixels. Because mosquitoes cannot see infrared light [35], we used two infrared light emitting lamps (Bosch Aegis SuperLed, 850 nm, 10° beam pattern (SLED10-8BD)) as illumination for the camera system. The camera system was calibrated at the start of each experimental day, with the use of a modified direct linear transformation (DLT) algorithm [36], by comparing known {*x*, *y*, *z*} coordinates of 25 suspended lead beads to their detected pixel coordinates in each camera. In addition, lens corrections were applied using pictures of a chequerboard pattern and using a Matlab script [37].

For each trial, 10 female mosquitoes were released from a holding container within the flight arena, after which the experimenter immediately left the room. From outside the room, the experimenter then started the 15 min video recording, and thus all experiments were performed without a potentially disturbing human present in the room. Five minutes after each trial, captured and non-captured mosquitoes were collected and killed. Out of the 61 successfully performed trials, a total of 53 trials were analysed (32 for the hanging trap and 21 for the standing trap). Five trials were discarded because male mosquitoes were found in the arena or trap and three other trials were discarded due to illumination or calibration errors.

### 2.3. Simultaneous tracking of multiple flying mosquitoes

To compute the three-dimensional tracks (flight segments) from the stereoscopic recordings of flying mosquitoes, we first determined the two-dimensional positions of the mosquitoes within each image using the image processing toolbox of Matlab (MathWorks). Then, for each camera recording, the two-dimensional flight tracks were constructed using a ‘Hungarian linker’ algorithm [38]. This algorithm reconstructs the tracks by finding the minimum distance between the detected positions of the mosquitoes in subsequent frames. Missed detections were taken into account by keeping tracks alive for 10 frames before deciding that they had ended. In the rare event that two tracks merged and then split up, two new tracks were started.

Next, the two-dimensional tracks within each camera view were combined into three-dimensional tracks using a DLT method [36]. A DLT error was calculated as the root mean square of the error (RMSE) between the original time-overlapping two-dimensional tracks and the two-dimensional back projections of the three-dimensional reconstructed tracks. To find the correct matches between two-dimensional tracks, we used an RMSE threshold that separated RMSE distributions for matching and non-matching tracks.

Finally, pieces of three-dimensional tracks were stitched together whenever a single two-dimensional track matched multiple two-dimensional tracks in the other image. A Hampel filter was added to remove positional outliers on the three-dimensional tracks. In this way, the complete flight track of a mosquito could be reconstructed. Individuals were, however, not identified because mosquitoes could enter and exit the filmed volume multiple times during one experiment. Throughout each resulting three-dimensional flight track, we calculated the mosquito's linear and angular flight speeds, and its linear acceleration (electronic supplementary material, figure S1). The angular flight speed was calculated as ω =Δθ/Δt, where $\Delta t=t_{n}-t_{n+1}$ is the time elapsed between two consecutive video frames *n* and *n* + 1, and Δθ is the turn angle defined as

2.1 $$\Delta \theta =\;\tan^{-1}\frac{\left|v_{n}\times \;v_{n+1}\right|}{v_{n}\;.\;v_{n+1}},$$

where $v_{n}$ and $v_{n+1}$ are the three-dimensional velocity vectors of the mosquito at video frames *n* and *n* + 1.

### 2.4. Analysing three-dimensional flight tracks

**Figure 3.**
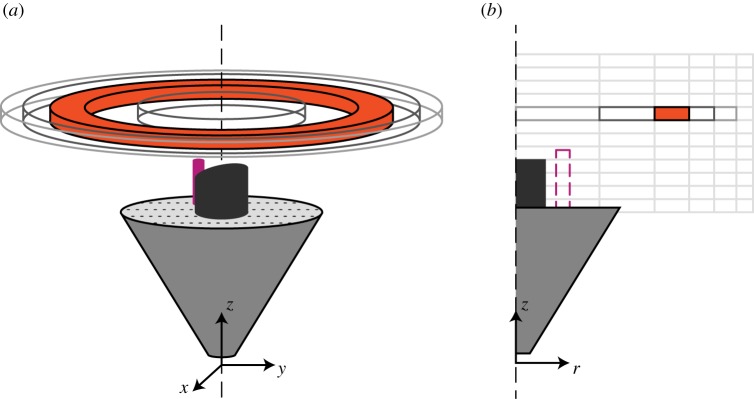
Visualization of three-dimensional flight dynamics in two-dimensional heat maps. (*a*) The filmed volume above the trap was divided into 1012 three-dimensional rings of equal volume, centred around the symmetry axis of the trap. (*b*) Various flight dynamics metrics were computed based the measured mosquito track dynamics inside each ring, and projected onto a two-dimensional parametric space comprising the vertical position (*z*-axis) and the radial position (*r*-axis) of each ring.

Owing to the high number of reconstructed tracks, a statistical approach was needed to visualize the flight dynamics of the mosquitoes around the two traps. For this purpose, we assumed the average flight behaviour of the mosquitoes around the trap to be axially symmetric, despite the presence of the trap's CO_2_ pipe and the slope of the trap's entry tube (visible in figure 3*a*). We divided the filmed volume into multiple three-dimensional rings (figure 3*a*), which were centred around the trap's axis of symmetry. We computed statistical metrics from the mosquito's position over time in each of the rings. To allow metric comparisons across the rings, the volume of all rings was the same. We then projected each three-dimensional ring onto a two-dimensional parametric space with radial distance and vertical position as the key dimensions (figure 3*b*). Similarly, top-down view projections were reconstructed by dividing the flight volume into three-dimensional vertical rods projected onto a two-dimensional horizontal plane.

Using this method, each flight dynamics metric was visualized as a set of two-dimensional heat maps, one on the radial–vertical plane and one on the horizontal plane. In addition to the translational speed, angular speed and acceleration, we also visualized the distribution of positional likelihood and capture probability of mosquitoes around the trap. The estimated capture probability was expressed by the percentage of mosquito tracks in each sub-volume that ended in capture. We defined the positional likelihood as the normalized probability of a mosquito to fly within a certain sub-volume (e.g. a specific three-dimensional ring) of the field of view. It was calculated as

2.2 $$P_{i}=\frac{n_{i}}{N}\cdot I,$$

where *P_i_* is the likelihood that a mosquito is present in cell *i*, whereby cell *i* represents a three-dimensional ring projected onto the previously described two-dimensional parametric space. *n_i_* is the number of video frames that a mosquito was present in cell *i* throughout all sets of recordings, *N* is the total number of frames recorded, and *I* is the total number of cells within the recording volume. Thus, a random flight behaviour would result in a uniform probability throughout the region of interest, with *P_i_* equal to one for all cells.

Furthermore, we visualized the mean flight dynamics as velocity vectors within the axisymmetric radial–vertical plane. Based on the resulting velocity fields, we visualized the average flight paths of mosquitoes by computing streamlines based on their velocity fields using the linear integral convolution algorithm [39].

**Figure 4.**
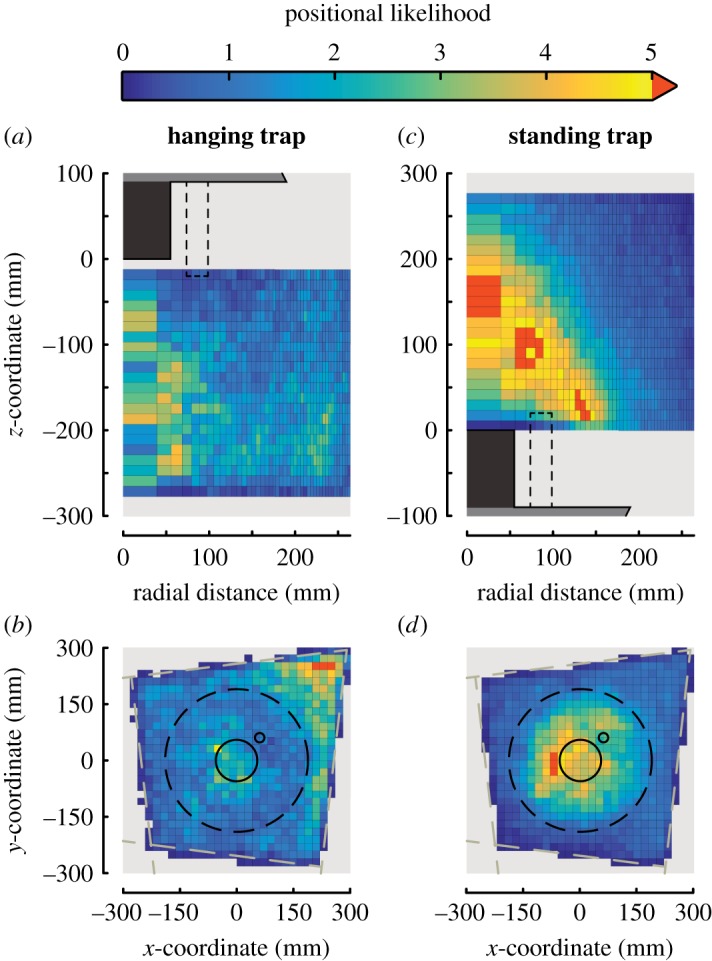
The spatial distribution of mosquito activity around the two traps, expressed by the positional likelihood. The positional likelihood, *P_i_*, was defined as the normalized probability of a mosquito to fly within a certain three-dimensional ring of the field of view. Random flight behaviour would result in a uniform probability throughout the region of interest, with *P_i_* equal to one for all cells. (*a,b*) The radial–vertical heat map (*a*) and the horizontal heat map (*b*) of the positional likelihood of all mosquitoes flying around the hanging trap, as indicated by the colour bar on the top. (*c,d*) Equivalent data for mosquitoes flying around the standing trap. (*a,c*) The solid black rectangle represents the trap entry tube, and the dashed rectangle indicates the radial distance at which the CO_2_ pipe was present. (*b,d*) The dashed circle, the large and small solid circles represent the circumference of the trap platform, the entry tube and the CO_2_ pipe, respectively.

**Figure 5.**
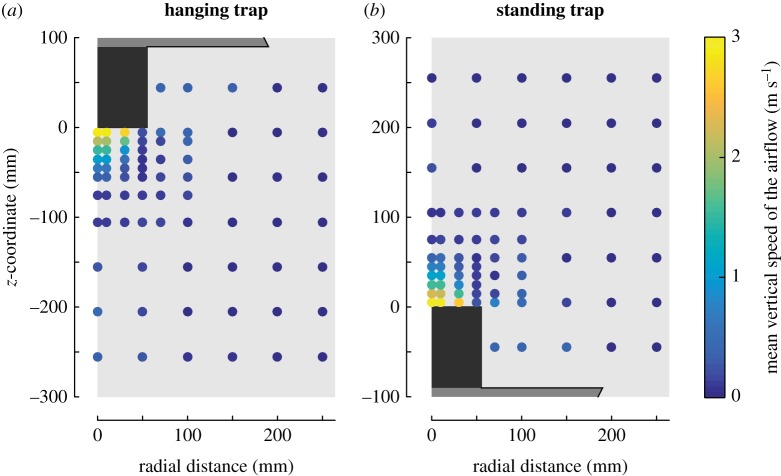
The vertical air speeds produced by the trap. The vertical air speed at 76 points around the hanging trap (*a*) and the standing trap (*b*), as colour-coded according to the colour bar to the right. See the electronic supplementary material, figure S5, for more details.

**Figure 6.**
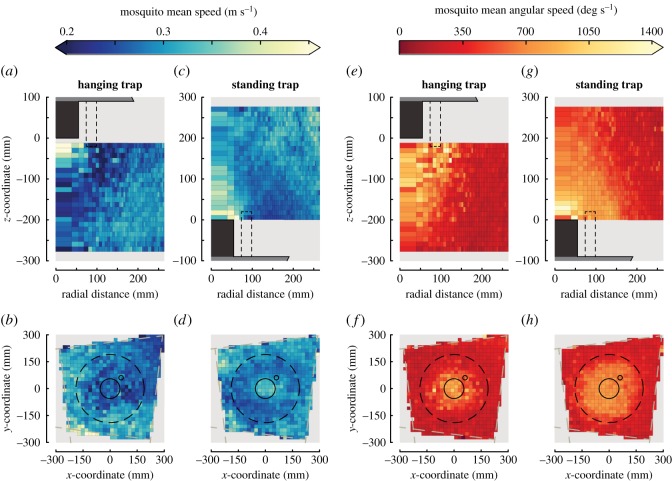
The average translational flight speed (*a–d*) and angular flight speed (*e–h*) of mosquitoes flying around the two traps. Panels (*a,c,e,g*) show the vertical–radial heat maps, and panels (*b,d,f,h*) show heat maps in the horizontal plane. (*a–d*) The average translational flight speed relative to the ground of mosquitoes flying around the hanging trap (*a,b*) and standing trap (*c,d*) are scaled according to the colour bar above (*a,c*). (*e–h*) The average angular flight speed of mosquitoes flying around the hanging trap (*e,f*) and standing trap (*g,h*) scaled according to the colour bar above (*e,g*). Standard errors and track number heat maps are shown in electronic supplementary material, figure S6.

**Figure 7.**
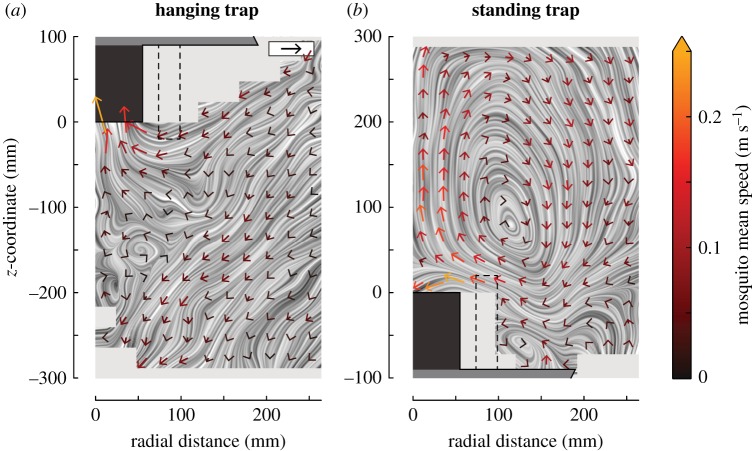
Averaged velocity vector fields and streamlines of mosquitoes flying around the hanging trap (*a*) and the standing trap (*b*). Each vector consists of the average velocity in the radial and vertical directions of all mosquitoes that flew within a cell of 24 × 24 mm. All velocity vectors that resulted from fewer than five detected tracks were discarded. Each velocity vector was scaled according to the black vector on the top right of panel (*a*) with magnitude 0.25 m s^−1^, and coloured according to the bar on the right. Standard errors and track number heat maps are shown in electronic supplementary material, figure S7.

**Figure 8.**
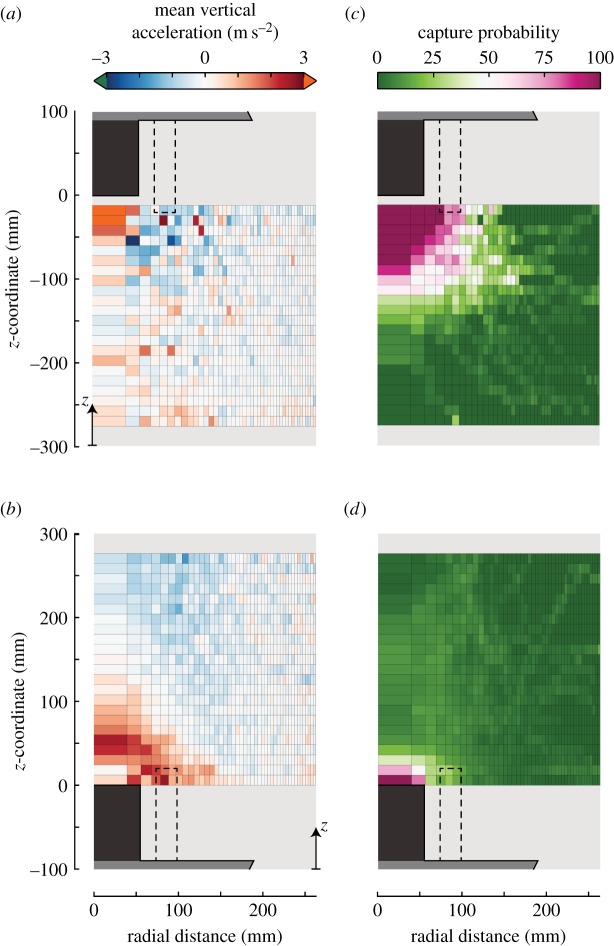
The distribution of average vertical accelerations of mosquitoes flying in the vicinity of the traps, and the distribution of the capture probability of mosquitoes flying around the traps. Panels (*a,c*) show data for the hanging trap, and panels (*b,d*) show standing trap data. (*a*,*b*) The average vertical accelerations of mosquitoes expressed by the vertical–radial heat map, as colour-coded according to the bar above (*a*). (*c,d*) Vertical–radial heat maps of the percentage of tracks that led to capture. The purple region represents the volume within which more than 75% of the tracks led to capture. Standard errors of the heat maps of (*a,b*) are shown in electronic supplementary material, figure S8. Top-down views of (*a–d*) are shown in the electronic supplementary material, figures S9 and S10.

For all calculated parameters, we performed sensitivity analyses to test the independence of our results to cell size, to the total number of analysed tracks and experimental duration (see electronic supplementary material, figures S2–S4). Additional data as well as 95% confidence intervals of the metrics presented in figures 5–8 are shown in the electronic supplementary material, figures S5–S10. For the statistical tests, we used a one-sample Kolmogorov–Smirnov test to determine whether data were normally distributed. Because all tested parameters were not normally distributed, we used the non-parametric Wilcoxon rank sum test to compare results between the hanging and standing trap (electronic supplementary material, table S1). We report results as *median* [*first quartile* – *third quartile*]. We used several Matlab colormaps from ColorBrewer [40] and from a published guide by Kovesi [41] to assign a unique colormap for each visualized metric (figures 4–8).

### 2.5. Analysing the airflow dynamics around the mosquito trap

The path of a mosquito flying around a trap results from a combination of the mosquito's manoeuvre dynamics and the air movements induced by the trap. Thus, to determine the effect of air movements on mosquito flight dynamics, we measured the vertical speed component of the airflow using a one-dimensional hotwire anemometer (tetso 405i). This device had a 1 Hz sample rate, and thus did not allow us to quantify turbulence levels. For these measurements, we again assumed the axial symmetry of the airflow, and thus ignored possible local differences in airflow around the CO_2_ pipe or due to the asymmetric design of the Suna tube entrance (see positional likelihood around the CO_2_ tube in electronic supplementary material, figure S11). Horizontal air velocities were not measured, as we expect that these were much lower than the vertical velocities induced by the vertically oriented fan.

With a custom-built set-up, we used the hotwire anemometer to measure the vertical airspeed at 76 locations within a two-dimensional vertical plane oriented perpendicularly to the open trapdoor in the trap entry tube and at the opposite side of the CO_2_ pipe. The standing trap was set at 59.2 cm above the floor, which was 3.7 cm higher than for the mosquito flight experiments. The hanging trap was kept at the same height as for the mosquito flight experiments (81 cm) because for this orientation, ground effects on the flow dynamics might be particularly important. Each velocity value was taken as an average over 45 s of measurements (at 1 Hz).

## 3. Results

### 3.1. Activity and capture rates of mosquitoes

We analysed 8 h of video recordings around the hanging trap, and 5 h 15 min around the standing trap. Despite the longer recording duration for the hanging trap experiments, only 897 mosquito tracks (pseudo-replicates) were detected around the hanging trap against 1673 tracks around the standing trap (electronic supplementary material, table S1). The number of flight tracks per trial (*N*_tracks_/*N*_trial_) for the standing trap was 2.7 times that for the hanging trap (hanging trap: *N*_tracks_/*N*_trial_ = 27.5 [20–33], *n* = 32 trials; standing trap: *N*_tracks_/*N*_trial_ = 75 [61.25–87.25], *n* = 21; *p* < 0.001). In addition, the duration of each track around the hanging trap was on average shorter compared with that around the standing trap (median track duration of 0.66 s and 0.99 s, respectively). Accordingly, the total flight duration per time recorded ((*∑T*_track_)/*T*_trial_) for mosquitoes flying around the standing trap was 5.1 times that for mosquitoes around the hanging trap (hanging trap: (*∑T*_track_)/*T*_trial_ = 1.53 [1.18–1.91]; standing trap: *∑*(*T*_track_)/*T*_trial_ = 7.84 [6.43–9.05]; *p* < 0.001). All these metrics show that flight activity around the hanging trap is lower compared with the activity around the standing trap.

Of all the 2570 recorded flight tracks, only 87 tracks resulted in capture of the mosquito by the traps, divided into 25 captures by the hanging trap and 62 by the standing trap. Because of the low number of tracks that led to capture, we were unable to compare the flight dynamics of the captured and non-captured tracks using our two-dimensional parametric space. Instead, to determine what causes the differences in number of captures between the traps, we calculated three different capture ratios: (i) the percentage of released mosquitoes that were captured (*R*_mosquitoes_ = *N*_captures_/*N*_released_·100%); (ii) the percentage of recorded tracks that led to capture (*R*_tracks_ = *N*_captures_/*N*_tracks_·100%) and (iii) the number of captures per minute flight duration (capture frequency *f*_captures_ = *N*_captures_/*T*_tracks_).

On average, only 8% of the mosquitoes released were captured by the hanging trap, whereas 30% of the mosquitoes were captured by the standing trap (electronic supplementary material, table S1). Therefore, the standing trap captured almost four times the percentage of released mosquitoes captured by the hanging trap. However, because there were many more flight tracks around the standing trap, the average percentage of flight tracks that led to capture by the standing trap was only 1.4 times higher than that for the hanging trap (electronic supplementary material, table S1). Because track duration was also longer for the standing trap, the number of captures per minute flight duration was not significantly different between the hanging and the standing trap (median of hanging trap: *f*_captures_ = 0 [0–3] min^−1^; median of standing trap: *f*_captures_ = 1.18 [0.96–1.98] min^−1^; *p* = 0.35). Thus, the hanging trap captured fewer mosquitoes than the standing trap. This difference can be explained by the higher activity of mosquitoes flying around the standing trap, which resulted in more and longer flight tracks around this trap.

### 3.2. Positional likelihood of mosquitoes

Heat maps of the positional likelihood of mosquitoes flying around the traps allow the identification of regions with increased activity, which are quite different between the two trap orientations (figure 4). Above the standing trap, a cone-shaped region of increased activity is visible, in which mosquitoes were up to five times more likely to be present compared to the average. By contrast, around the hanging trap no such clearly defined region of increased activity was found, except for a smaller, cylindrical region directly underneath the entry tube edge within which mosquitoes are up to four times more likely to be found.

The top-down views (figure 4*b,d*) show that flight activity is approximately axially symmetric, except for a small reduction in activity near the CO_2_ pipe (see also electronic supplementary material, figure S11), and for the hanging trap an interesting concentration of mosquitoes near the bottom corner of the back walls of our experimental set-up was present (figure 1).

### 3.3. Airflow dynamics of the traps

We visualized the airflow dynamics around the standing and hanging trap as the two-dimensional distribution of vertical speeds (figure 5). The distribution of air speed around the traps was consistent with previous findings [25], where vertical airflow speeds were very high at the mouth of the entry tube (up to 3 m s^−1^), but these speeds rapidly decreased when moving away from the trap entrance. As a result, the region in front of the tube where the airflow is more than 1.5 m s^−1^ is only 3 cm high and 10 cm wide. This suggests that for a mosquito to be inevitably captured by the inward-directed airflow, it needs to pass very close to the trap entrance. A comparison of the vertical airspeeds around the standing and hanging trap (figure 5 and electronic supplementary material, S5) shows that trap orientation has a relatively small effect on airflow dynamics, suggesting that a possible ground effect below the hanging trap is mostly negligible.

### 3.4. Mosquito flight dynamics

Based on all flight trajectories, we determined the translational flight speeds, angular speeds and accelerations of mosquitoes around the hanging and standing trap (electronic supplementary material, figure S1). The mosquitoes flying around the standing trap flew faster, had higher turn rates as expressed by higher angular speeds, and they produced higher body accelerations than those around the hanging trap (*p* < 0.001; see electronic supplementary material, table S1).

The two-dimensional distributions of the translational speeds of mosquitoes flying around the standing and hanging traps were similar (figure 6*a*–*d*). Mosquitoes had their highest mean ground speeds (up to 0.5 m s^−1^) near the trap entrance, whereas in both trap orientations they had their lowest mean speeds at a radial distance of 10–20 cm from the entry tube. At both these regions with increased and reduced translational speeds, the angular speeds were high (figure 6*e–h*).

In contrast with the relatively high similarity in absolute translational and angular flight speeds around the traps, the velocity fields around the traps were strikingly different (figure 7). The pattern of mosquito streamlines near the hanging trap suggests that two regions can be identified, separated by a diagonal that runs from the top right to the bottom left of the velocity field. On average, mosquitoes that flew below this diagonal continued to fly downwards and away from the trap, whereas those flying above this border would initially fly downward, but when they got close to the trap entrance, they turned towards the trap entrance and got caught. Example flight tracks of mosquitoes within these two regions are in figure 2*a* and electronic supplementary material, movie S1.

Around the standing trap, mosquitoes followed a more complex circulating flight dynamic. On average mosquitoes entered the filmed volume by flying down towards the trap platform, after which they turned towards the black entry tube. When they were above the tube, mosquitoes either got caught by entering the trap, or they escaped capture by accelerating upwards. After flying upwards for approximately 20 cm, on average they turned around and started to fly again downwards towards the trap platform, thus completing the loop. Two typical examples of mosquitoes performing such a circulating flight manoeuvre can be seen in figure 2*b* and electronic supplementary material, movie S2.

As for the hanging trap, we also identified the capture and escape regions for the standing trap based on the distributions of mosquito streamlines. The area for which the streamlines end at the trap entrance is much smaller for the standing trap than for the hanging trap, suggesting that mosquitoes need to approach the standing trap entry tube more closely before being captured than for the hanging trap.

As expected, accelerations of mosquitoes flying around the trap were highest near the trap entrance (figure 8*a,b* and electronic supplementary material, figure S6). Around both traps, the mosquitoes flying close to the tube entrance tended to accelerate upwards, despite the oppositely oriented airflow for capture of the different traps. This means that the mosquitoes flying near the entry tube of the standing trap, accelerated in the direction opposite to the airflow direction, and thus avoided capture. On the other hand, the mosquitoes flying near the hanging trap also accelerated upwards, but in this case, it was in the same direction as the airflow, and thus these mosquitoes flew straight into the mouth of the trap.

### 3.5. Distribution of the capture probability

The distribution of the capture probability is remarkably different for mosquitoes flying around the two oppositely oriented traps (figure 8*c,d*). Mosquitoes that approached the entry tube of the hanging trap within a 10 cm radius have a 75% chance of being caught, whereas for the standing trap mosquitoes need to enter the single cell directly above the entry tube for the likelihood of being caught to reach 75%. We reconstructed the three-dimensional volume within which more than 75% of detected mosquito tracks resulted in captures by revolving the two-dimensional heat map around the axis of symmetry. For the hanging trap, this volume was 17.5 times larger than for the standing trap (electronic supplementary material, table S1).

## 4. Discussion

We reconstructed three-dimensional flight tracks from stereoscopic high-speed videos of mosquitoes flying around an odour-baited trap set in two different orientations. The resulting high number of detected tracks was essential to dissect the flight behaviour of mosquitoes around the traps as it allowed the computation of the average flight dynamics throughout the complete region of interest. To study how these dynamics vary in space and as a function of trap orientation, we visualized these three-dimensional flight dynamics using two-dimensional heat maps and vector fields. For this, we assumed that the average flight behaviour of mosquitoes around the trap was axisymmetric with respect to the axis of symmetry of the trap, which was confirmed as almost all top-down heat maps showed exclusively axisymmetric patterns.

The only deviation from this symmetry was an off-centred volume under the hanging trap within which mosquitoes had a high probability of being present (figure 4*b,d*). Mosquitoes might have been attracted to this region because of an accumulation of odours and CO_2_ in this corner, or because a shadow was cast here by the hanging trap. But because top-down views of all other metrics presented in this paper did not show a bias towards this area, we cannot conclusively determine the cause of this accumulation. Additionally, we observed a small reduction in activity near the CO_2_ pipe of both traps (figure 4*b,d* and electronic supplementary material, figure S11*a*), suggesting that mosquitoes were avoiding this region, possibly due to increased CO_2_ concentrations or airflow anomalies near the shortened CO_2_ pipe [7,9,42,43]. Note that both the increased activity of mosquitoes at the off-centred volume under the hanging trap and the reduced activity near the CO_2_ pipe had a relatively small effect on the flight pattern of mosquitoes in the near vicinity of the trap (figure 6 and electronic supplementary material, figure S11*b*, respectively), suggesting that the assumption of axisymmetric flight behaviour around the trap is still valid.

Beside these small anomalies, the heat maps of positional likelihood of the mosquitoes (figure 4), and of mean flight speed and mean angular speed (figure 6) contribute some new elements concerning the short-range attractiveness of the studied odour-baited traps. Indeed, with the exception of the previously described regions, the volumes where mosquito activity was the highest are also where their mean flight speeds and mean angular speeds were the highest, suggesting that these mosquitoes were performing casting behaviour and might thus have been host-seeking. This is consistent with findings of a previous study whereby host-seeking mosquitoes increased their flight tortuosity in the proximity of a host [10]. Given that the used odour-baited trap has been shown to be successful in attracting host-seeking mosquitoes [25,26], we hypothesize that the majority of mosquitoes flying close to the traps were attracted by the trap, despite that random encounters with the traps likely also occurred.

The question remains as to what particular sensory cues trigger the search behaviour within the highly unsteady airflow conditions around the trap. For example, mosquitoes have been found to surge upwind more easily in airflow well mixed with odours and in turbulent CO_2_ plumes [44,45]. To answer this question, an extensive study of the airflow turbulence levels and three-dimensional distributions of CO_2_ and odour would be required [46].

### 4.1. Stereotypical mosquito flight dynamics

The flight dynamics of mosquitoes around the two oppositely oriented traps were strikingly different but also highly stereotypical and repeatable (figure 7). Near the hanging trap set-up, mosquitoes flew on average down towards the ground. But if they flew within a range of approximately 10 cm from the trap entry tube during this downward flight, they would turn and start to fly upwards, and as a consequence likely be caught by the trap. Mosquitoes flying around the standing trap showed a very different, circular flight pattern. These mosquitoes would initially also fly downwards when entering the filmed volume, but in this case towards the trap platform instead of the ground. When they came close to the entry tube, they would also turn and start to fly upwards, which resulted in most cases in a successful escape from the trap. After this manoeuvre, they would turn around and again start to fly downwards towards the trap platform, completing the circular flight pattern. For the standing trap, mosquitoes had to approach the entry tube much closer in order to be captured than for the hanging trap (figure 8*c,d*).

Because the trap-induced airspeeds were very similar between the traps (figure 5; electronic supplementary material, S5), the large differences in flight dynamics around the two traps must be the result of a difference in behavioural response towards the two oppositely oriented traps. Here, we hypothesize that these flight patterns are the result of two stereotypic behaviours in host-seeking mosquitoes: (i) mosquitoes that approached a trap tended to also fly downwards to the ground and (ii) mosquitoes that came close to the traps changed their flight direction by rapidly accelerating upwards, possibly reacting to adverse high-velocity airflow cues or to a lack of short-range host cues. This set of behaviours can explain the complex flight patterns observed around the traps, as well as the differences in flight patterns around the oppositely oriented traps.

#### 4.1.1. Upwind- and downward-directed approach flights

Upon entering the observed volume and despite inverse trap airflow, mosquitoes flew on average downwards, as well as towards the axis of symmetry of the trap, for both traps (figure 7). While doing so, they had low angular speeds and low positional likelihood (figure 4). Thus, mosquitoes did not remain long in the flight volume for this approach phase. As a result of the downward orientation, mosquitoes naturally flew toward the standing trap, whereas they tended to fly away from the hanging trap.

This flight behaviour is very similar to that described in female mosquitoes seeking a human host. Female *Anopheles* mosquitoes preferred to land on human body parts that were closest to the ground, which they found by flying downwards and upwind by tracking odours and convective air currents produced by that host [30,47]. When approaching an odour source, *Aedes aegypti* mosquitoes flew towards the ground to inspect visually intriguing objects [9,18]. The similarity in flight pattern of our mosquitoes flying near the trap to those described in the literature suggests that our mosquitoes performed a similar host-seeking behaviour. In our case, the upwind and downward flight behaviour might have been triggered by the detection of increased concentrations of odours, CO_2_ and airflow turbulence, but the importance of visual cues cannot be excluded as possible light conditions differences have not been investigated.

#### 4.1.2. Fast upward-directed flight manoeuvres

The second phase of mosquito flight dynamics takes place in the near vicinity of the trap entry tube. It starts at the boundary between the high-speed airflow volume a few centimetres from the visually contrasting entry tube and the volume where attracted mosquitoes are host-seeking. Here, on average, mosquitoes were found to quickly turn by accelerating and flying upward. For the mosquitoes that flew below the hanging trap, these rapid upward flight manoeuvres often led to capture, whereas the mosquitoes that flew above the standing trap, accelerated away from the trap and thus mostly escaped successfully.

Similar fast upward-directed flight behaviours have been described previously for other host-seeking insects [18,31,32]. Host-seeking *Anopheles* mosquitoes tended to fly quickly upwards after inspecting black tiles on the ground [18], and horse and deer flies tend to fly upwards after having inspected potentially interesting visual cues [31,32]. In fact, the Malaise trap and the Manitoba fly trap have both been designed to trap such upward flying insects [31,32].

The question remains about what sensory cues trigger these rapid upward flight manoeuvres near the trap. This could be the absence of short-range host cues such as heat or moisture [17,18], or the flight manoeuvre could be the result of positive phototaxis, reaction to visual cues, or to avoid/evade the high-speed airflow regions. The upward flying mosquitoes in the shadow of the hanging trap flew towards its black entry tube, whereas mosquitoes flew away from the black entry tube of the standing trap. Therefore, positive phototaxis as well as reaction to visual cues most likely do not explain these manoeuvres. Thus, these upward manoeuvres were probably either the result of the absence of short-range host cues, or they might have been executed to avoid or evade the high-speed airflow regions induced by the traps.

Should these upward-directed flights be evasive manoeuvres, then mosquitoes did not use information about the direction of the airflow to steer away from a potential threat, as all manoeuvres were directed upwards regardless of the direction of the airflow. This makes these manoeuvres strikingly different from the evasive manoeuvres described in flying fruit flies, hawkmoths and hummingbirds, that are directed away from the danger [48,49], or downwards towards the ground [50]. Horse flies, on the other hand, have also shown to fly upwards after inspecting potential hosts [31,32]. Because mosquitoes and horse flies both feed on terrestrial animals, these upward-directed evasive or avoidance manoeuvres might be particularly successful for such animals.

The stereotypical upward accelerating manoeuvres of our mosquitoes also explain the large difference in capture dynamics between the oppositely oriented traps. The air volume around the trap entrance within which 75% or more of the mosquitoes were caught, *V*_75%_, was 17.5 times larger for the hanging trap than for the standing trap (figure 8*c,d*). For the standing trap, this *V*_75%_ volume overlays well with the region within which the airspeeds directed into the entry tube were more than 1.5 m s^−1^ (figure 5), but for the hanging trap the *V*_75%_ volume extended well outside this high airspeed region. In flow tunnel experiments, it has been demonstrated that mosquitoes are able to fly against airflow with speeds of up to 1.5 m s^−1^ [51], and thus mosquitoes caught by the standing trap were most likely sucked downwards into the trap, as they were unable to accelerate upward fast enough to avoid capture. Because the *V*_75%_ volume underneath the hanging trap extended well beyond the region with high suction airspeeds, many of the mosquitoes captured by the hanging trap must have actively flown into the entry tube when performing an upward-directed flight manoeuvre.

#### 4.1.3. Combining the two stereotypical flight behaviours

**Figure 9.**
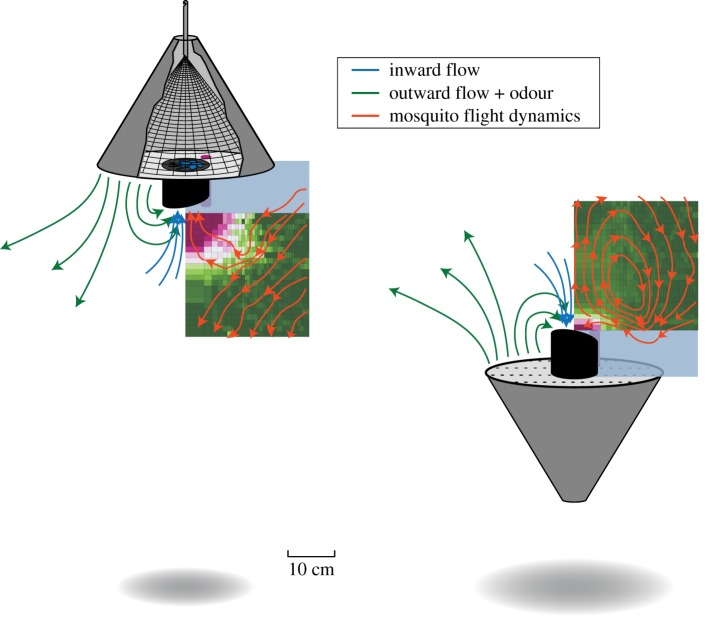
The average flight tracks and capture percentage of mosquitoes around the two traps, and an artist impression of airflow induced by the traps. The mean flight tracks (in red) are determined from the streamlines in figure 7, and details about the capture percentage distribution (heat map) are shown in figure 8*c,d*.

The flight dynamics observed near the two traps can be interpreted as a combination of the upwind- and downward-directed approach flights and the fast upward-directed evasive manoeuvres. Although alternative behavioural explanations of the observed flight dynamics are possible, the here-described flight behaviours have previously been identified in host-seeking insects [9,18,30–32], and the combination of these two behaviours explains well the striking differences in flight dynamics around the oppositely oriented traps (figure 9). The upwind- and downward-directed flight behaviours illustrate why mosquitoes tended to fly away from the hanging trap, and towards the horizontal platform of the standing trap; the fast upward-directed flight manoeuvres caused mosquitoes to fly towards the capture entrance of the hanging trap and away from the standing trap, and thus also explain the larger capture region around the hanging trap (figure 8). After the upward flight movement away from the standing trap, these mosquitoes switched back to the downward-directed flight pattern, explaining the advent of the circular flight path, and why mosquitoes remained near the standing trap for a longer time.

### 4.2. The flight behaviour of mosquitoes explains trap efficiency

Our results suggest that the standing trap has a higher short-range attractiveness, as expressed by both a higher number of detected flight tracks (*N*_track_/*N*_trial_) and larger flight duration per trial (*T*_track_/*T*_trial_) for mosquitoes flying around the standing trap. By contrast, the hanging trap has a better capture mechanism because the air volume around the trap entrance within which 75% or more of the mosquitoes were caught, *V*_75%_, was more than 17 times larger for the hanging trap compared with the standing trap. However, both the percentage of released mosquitoes caught and the percentage of flight tracks that led to capture (*R*_mosquitoes_ and *R*_tracks_, respectively) were higher for the standing trap. Although *R*_mosquitoes_ and *R*_tracks_ were not significantly different between the traps, this suggests that the less efficient capture mechanism of the standing trap was more than compensated by its superior short-range attractiveness. In this way, the greater number of mosquitoes captured by the standing trap seems to be not only because it attracts more mosquitoes to its vicinity, as was previously suggested [5], but also because mosquitoes remained near the standing trap for a longer period of time.

These results, and especially the differences in capture efficiency, would need to be verified in field experiments, where the wind and light conditions would likely impact trap finding by the mosquitoes as well as their use of visual cues. In addition, the fact that our experiments were performed in an enclosed environment, where the proportion of random encounters might have been relatively high, may have impacted our results. However, such random encounters would probably only increase the background noise on the observed flight dynamics and should not result in distinct flight patterns. Finally, height differences might have affected how easily mosquito were finding the trap, and hence the trapping efficacy [25]. This subject would deserve to be studied in dedicated experiments.

Besides contributing to the expansion of general knowledge on mosquito flight behaviour, our results help to understand the short-range attractiveness and the capture mechanism of odour-baited traps. This new insight could be used to develop novel trap designs with improved trapping efficiency. Thus, in our opinion, such trap design process would greatly benefit from the use of iterative testing of traps in studies similar to this one. Because the Suna trap is already part of a successful vector control system [26], it is likely that the resulting trap improvements would make a valuable contribution to the fight against vector-borne disease.

## Supplementary Material

Supplementary material

## Data accessibility

The datasets supporting this article are available in electronic supplementary material, databases S1 and S2.
